# Risk Factors For Insomnia Among Care Partners Recruited From An Outpatient Memory Care Clinic

**DOI:** 10.1002/alz.086320

**Published:** 2025-01-09

**Authors:** Virginia Gallagher, Meghan K Mattos, Ashleigh Patterson, Ryan C Thompson, Shannon Reilly, Ishan Canty Williams, Kelly M Shaffer, Carol A Manning

**Affiliations:** ^1^ University of Virginia, Charlottesville, VA USA

## Abstract

**Background:**

Prior research on factors associated with sleep problems among care partners (CPs) of persons with cognitive decline (PwCD) are often limited by imprecise (i.e., single yes/no questions) measures of insomnia, burden, and CP mental health. This study aimed to: 1) determine the prevalence of insomnia disorder using DSM‐5 criteria among PwCD CPs in an outpatient memory care clinic, and 2) identify risk factors for CP insomnia symptoms using rigorous measures. We hypothesized that caregiver burden, depression, and role overload would be the strongest predictors of PwCD CP insomnia symptoms.

**Methods:**

In this cross‐sectional survey study, PwCD CPs completed a demographic and caregiving questionnaire; a modified Insomnia Severity Index (ISI) that included assessment of frequency, duration, and impact of insomnia symptoms per DSM‐5 insomnia disorder criteria A‐D; Short Form Zarit Burden Interview; and the Hospital Anxiety and Depression Scale. Multiple linear regressions using forward elimination were used to determine the most robust predictive model of PwCD CP insomnia symptoms (ISI).

**Results:**

Among all CPs (n = 80), 10.00% met DSM‐5 insomnia criteria A‐D. Regarding ISI score classification, 26.00% CPs had clinically significant symptoms of insomnia (ISI≥10). The most robust risk factor model [R^2^ = .423, F(3,76) = 18.605, p<.001] for CP insomnia symptoms included CP anxiety (β = .374, p = .004), CP‐PwCD co‐dwelling status (β = ‐.229, p = .012), and burden (β = .255, p = .042), and did not include factors such as demographics and PwCD nighttime needs and behaviors. Among CPs who co‐dwelled with the PwCD (n = 65), the most robust risk factor model [R^2^ = .412, F(2,62) = 21.764, p<.001] for CP insomnia symptoms included CP burden (β = .392, p = .004) and CP anxiety (β = .309, p = .022), and did not include other factors.

**Conclusions:**

CP mental health symptoms and caregiving burden appear to be notable risk factors for PwCD CP insomnia symptoms, which has implications for intervention approaches for CPs.

